# Supplementary material for: Are carbon emissions trading and green financial instruments synergistic? -Comprehensive quantitative research based on content analysis
Source: PLoS One. 2024 Mar 7;19(3):e0298601. doi: 10.1371/journal.pone.0298601 (PMC10919601; doi:10.1371/journal.pone.0298601)
Supplement: S1 Data — (ZIP) [file pone.0298601.s001.zip › Data/地级市Z_F工作报告.zip!/║■▒▒/╢⌡╓▌╩╨/└┤╫╘╣1⁄2╓┌║┼í╛┬φ┐╦╩2╛▌═°í┐_2009.docx]

各位代表：

　　现在，我代表市人民政府向大会作政府工作报告，请予审议，并请各位市政协委员和列席人员提出意见。

　　一、2008年工作回顾

　　2008年是极不平凡的一年。面对历史罕见的雨雪冰冻等严重自然灾害和严峻复杂的国际国内经济形势，全市上下深入贯彻落实科学发展观，按照省委、省政府和市委的决策部署，冷静研判，坚定信心，对宏观政策调整和外部环境变化保持高度敏锐，快速及时制定应对政策，在推进项目建设、力保工业增长、扶持中小企业等方面采取了一系列强有力的措施。经过艰苦努力，全市经济平稳较快增长，各项社会事业全面进步。实现地区生产总值269.79亿元，同比增长15.8%，在全省居第二位；财政总收入25.31亿元，增长20.8%；地方一般预算收入10.7亿元，增长25.8%；固定资产投资150亿元，增长42.8%；招商引资总额64亿元，增长42.2%；实际利用外资9336万美元，增长15.6%；全社会消费品零售总额104.9亿元，增长24%；城镇居民人均可支配收入12244元，增长13.1%；农民人均纯收入5096元，增长16%；单位生产总值综合能耗下降4.5%，化学需氧量、二氧化硫排放量分别下降2.7%和7%。除外贸出口受宏观形势影响未达目标外，本届人大第一次会议确定的2008年主要预期目标均超额完成。

　　——奋力抗击雨雪冰冻灾害，农村经济持续稳定发展。年初发生特大雨雪冰冻灾害后，及时启动应急动员机制，全力做好应急管理、对口帮扶、农业抗灾、科技支农等工作，最大限度减小灾害造成的损失。切实加大了财政投入力度，支农支出达到2.44亿元，比上年增长70.2%，是历史上支农资金最多的年份。农产品产量在大灾之年平稳增长，实现农业总产值60.79亿元，增长8.4%。城乡一体化扎实起步，省委、省政府正式批准鄂州为全省城乡一体化试点城市，编制完成了鄂州城乡总体规划和城乡一体化规划纲要，投入6000万元建设特色镇基础设施，中心村建设试点开始启动。深入开展“清洁乡村、美化家园”活动，农村生产生活条件进一步改善，10.2万农村群众喝上了自来水，改造堰塘328口，更新中小泵站74座，完成渠网硬化100公里；新增通村公路146公里，新建沼气池3600口，同步“一池三改”3600户；建成高产农田7万亩；培养农民科技示范户2000户，机械化率达到46.6%；启动了第二轮30个重点村整村推进扶贫开发，农村面貌明显改观。

　　——有效应对全球金融危机，工业经济实现较快增长。面对突如其来的金融危机，主动应对、积极作为，针对危机给经济发展特别是工业企业生产经营带来的严重冲击，坚持与企业同舟共济、共克时艰，迅速出台支持中小企业发展的8项措施，筹资1000万元用于中小企业技术改造和高新技术产业化贷款贴息，开展银行行长“进企业、访客户、解难题”活动，落实项目、企业“直通车”和保姆式服务，大力整治企业生产和经营环境。工业增加值在危机之年突破百亿，达到126.5亿元，增长26.1%。重点产业对经济支撑带动作用进一步加强，冶金、装备制造、建材、纺织服装、医药、能源等支柱产业共完成增加值101.7亿元，增长26.46%；实现销售收入281.2亿元，增长33.67%。

　　——着力推进经济结构调整，发展后劲整体提升。围绕建设宜居宜业大城市的发展目标，从整体上谋划和推动产业结构调整。加快现代服务业和高新技术产业发展，梁子岛生态旅游区（梁子镇）成为全省首批旅游名镇，成功举办第九届梁子湖捕鱼旅游节，服务业共实现增加值80.18亿元，同比增长10.6%。加快高新产业发展，高新技术产业实现增加值25.19亿元，增长27.8%；高新技术产品出口1100万美元，增长69.2%。大力实施中小企业成长工程，全年新增规模工业企业103家，是建市以来最多的一年；销售收入过亿元的企业达到50家，同比增加21家；扩大小额贷款规模，总额达2936万元，居全省第三位；新发展个体工商户4430户，同比增长113.9%。大力实施品牌战略，实现商标国际注册零突破。进一步扩权强区，“两区三城”发展加快，完成基础设施建设投入5亿元，葛店开发区财政收入突破2亿元，鄂州经济开发区经省政府批准筹建，进入实质性运作，梁子湖生态旅游区、红莲湖旅游新城、花湖工贸新城发生较大变化。启动一批对鄂州长远发展有重大影响和带动作用的重大项目，全年有29个亿元以上项目开工；中央扩大内需促进经济增长的措施出台后，共向国家发改委申报33个国债项目，总投资2.48亿元，已有1.1亿元列入国家投资计划；梁子湖水系综合治理、武汉新港三江港区等48个项目正在积极申报中。

　　——加速推进基础设施建设，城市综合承载力不断增强。大力提升城市功能，主城区基础设施建设市财政投入5.5亿元，实现历史性突破。主城区道路刷黑、观音阁公园、吴都大道、洋澜湖截污等九大重点项目完成预期目标；对城区31条背街小巷进行了综合整治，对南浦游园二期、沿湖道路、凤凰路等8条路段实施了绿化改造；交通建设完成投资2.2亿元，汉鄂快速通道已开工建设，对接武汉的重点工程高新四路、六十至流芳一级公路、316国道华容段改造相继开工，葛湖线过渡路面、司燕线、燕沙线建成通车，鄂州中心客运站启用。加速推进“四城创建”，开展了城区交通环境等专项整治。深入推进城市管理体制改革，实施了城管执法进街道、监管进社区的管理体制，形成了“重心下移、属地管理、条块结合、以块为主”的管理模式，城市管理水平进一步提升。

　　——切实加大民生投入，社会事业全面进步。认真办理“十件实事”，共投入资金4.4亿元，受惠群众60多万人，年初承诺全部兑现。投资5000多万元建成吴都中学并按期开学，筹资760万元兴建和扩建8所农村寄宿制学校，农村学生实现免费义务教育，城区学生实现免学杂费入学，职业教育快速发展，葛店开发区“湖北职业教育园”建设取得积极成果。全年争取科技项目40项，申请专利414件，被国家知识产权局批准为全国知识产权试点城市。三国吴都博物馆动工建设，鄂州雕花剪纸被列入国家级非物质文化遗产名录，建成150个行政村农家书屋，在全省率先实现农民健身工程全覆盖，奥运主题群众文体活动蓬勃开展。争取国债资金3000多万元，完成了市紧急救援中心、传染病医院、疾控中心及14个乡镇卫生院三项建设和46个村卫生室建设。统筹城乡就业工作全面展开，创业促就业工作扎实推进，城镇新增就业19380人，下岗失业人员再就业6546人。社会保障体系逐步完善，筹集各项社会保险资金7.3亿元。新农合大幅扩面提标，筹资5600余万元，受益30万人次；启动城镇居民医疗保险，登记参保人数15万人，综合医疗保险覆盖率达87％。住房保障工程加快实施，投入1000多万元对农村危房彻底改建，新建经济适用房200套。发放住房公积金个人贷款1.46亿元，使1121户低收入家庭圆了住房梦。筹资2500万元，新建和改造了城市社区办公活动场所。顺利完成村委会换届选举。人口计生工作质量明显提高，人口自然增长率控制在5‰以内。积极开展抗震救灾，全市累计捐款1104万元，对口支援汉源县九襄镇灾后重建工作抓紧进行，汉源县第一中学等5个援建项目已全部开工。援疆工作按照计划顺利推进。

　　——努力改善发展环境，社会保持和谐稳定。全面构建依法行政体系，在全省率先启动以“职权法定、流程再造、绩效考评、行政问责”为主要架构的依法行政体系建设，制定了《市人民政府关于加强依法行政的决定》，发布了《鄂州市行政职权目录》，《鄂州市行政职权运行流程》开始试运行，议事决策规程全面落实，权力运行机制逐步完善。深入开展文明执法教育活动，粗暴执法、野蛮执法现象得到有效遏制。严格实行重大行政处罚备案审查制度，行政执法质量稳步提升。广泛宣传行政复议制度，行政监督力度明显加强。深入开展提高政府执行力活动，着力推进效能监察，服务环境不断优化。政务事务公开稳步推进，治理商业贿赂工作成效明显。审计力度不断加大，全年完成审计128项，查处各类违纪违规金额11979万元，挽回直接损失238万元。 自觉接受人大法律监督和政协民主监督，2008年共办理人大建议和政协提案391件，办复率100%，满意和基本满意率达到99.4%。加速推进“两型社会”先行区建设，编制完成了《总体方案》和《三年行动计划》。环保专项整治成效显著，市污水处理厂投入使用，葛店开发区污水处理厂投入试运行，鄂钢污水处理二期工程完成验收。进一步规范矿产资源管理，违法探矿采矿行为得到有效遏制。普法教育深入推进，社会治安综合治理得到加强，重大恶性案件得到有效遏制，命案告破率达100%。大力整顿市场秩序，工商行政管理、质量技术监督和食品、药品、烟草市场监管进一步加强。深入开展了重点行业和重点领域的安全生产隐患排查治理工作，安全生产保持平稳态势。信访工作得到加强，各级领导实行包案下访，有力地促进了社会稳定。

　　与此同时，统计、物价、粮食、供销、民族、宗教、外事、侨务、对台、档案、方志、工会、青年、妇女、儿童、残联、供电、保险、邮政、通讯、石油石化、国企改革、国资管理、无线电管理、广播电视、新闻出版、文学艺术、社会科学、人防、老龄、气象和国防教育、民兵建设等工作都取得了新的成绩。

　　各位代表，在严重自然灾害和全球金融危机之年，鄂州经济社会继续保持了良好发展势头，是经过艰苦努力和奋力拼搏取得的，成绩来之不易，这是市委正确决策和坚强领导的结果，是市人大、市政协和各民主党派、工商联及无党派人士关心和支持的结果，是全市人民团结奋斗的结果，也得益于历届市委、市政府打下的良好基础。借此机会，我谨代表市人民政府向辛勤工作在各个岗位上的广大干部群众，向各位人大代表、政协委员，向各民主党派、工商联、无党派人士、人民团体和社会各界人士，向为鄂州发展作出贡献的各位老同志，向驻军指战员、武警官兵以及所有来鄂州的投资者、劳动者和海内外朋友表示崇高的敬意和衷心的感谢！

　　在充分肯定成绩的同时，我们也清醒地认识到面临的困难和问题。一是全球金融危机和经济衰退的影响仍在进一步显现，投资增长减缓，企业效益下滑，经济发展对少数产业、行业、企业依赖性仍然过大，高新技术产业和现代服务业比重仍然偏低，民营经济规模不大，经济发展抗风险能力较弱；二是新农村建设任务艰巨，农业产业化龙头企业发展较慢，农民收入持续增长压力较大，农民生产生活条件改善任务较重，城乡一体化运行体制和工作机制亟待建立和完善；三是和谐鄂州建设仍然任重道远，进一步完善社会保障体系、维护社会公平正义、扶助弱势群体、实现安全生产的任务仍然相当繁重；四是政府自身建设尤其是在科学发展、依法行政、提升效能等方面还需要进一步加强和改进，等等。对这些问题，我们将进一步增强忧患意识和责任意识，采取更加有力的措施，认真加以解决。

　　二、2009年工作安排

　　2009年是建国60周年，是实现“十一五”发展规划的攻坚年，是深入开展学习实践科学发展观活动、促进科学发展上水平的重要一年，也是鄂州实现“两个率先”、建设“两型社会”的关键一年，做好今年的政府工作，具有十分重要的意义。

　　今年我们面临的经济形势异常复杂、异常严峻、异常困难。世界主要经济体仍可能继续衰退，新兴市场国家和发展中国家经济增速将明显放缓，外部需求减弱，势必加剧国内市场竞争，给企业发展带来更大压力。目前，金融危机正由沿海传导到中部，不仅本身尚未见底，而且对实体经济的影响进一步加深。总体上看，2009年可能是我市新世纪以来经济发展所面临的外部环境最为困难的一年。面对危机，消极对待，被动应付，危机就是困难，就是危局；知难而进，积极应对，危机就是动力，就是转机。在清醒认识当前形势的严峻性、复杂性的同时，更应看到变化和转换中存在着的有利条件，抓住困难和挑战中蕴藏着的重大机遇：第一，中央为应对国际金融危机采取了一系列得力有效的果断决策，引发危机的根本因素在我国被有效遏制。国家稳定的宏观经济大局，全省加速发展的良好势头，鄂州改革开放以来快速发展所打下的坚实基础，为我们实现平稳较快发展提供了坚强支撑。第二，国家出台扩大内需保发展的一系列政策措施，实施积极财政政策，为我们争取政策、争取项目、争取资金提供了有利时机；实施适度宽松的货币政策，这将降低企业特别是中小企业的融资门槛和融资成本。全面实施增值税转型改革，必将刺激企业增加技术改造和增产扩容投资，促进资本有机构成较高的重工业提升装备水平，加快产业升级，这对重工业比重高的鄂州来说，是一次加快转变发展方式、推动发展提档升级的难得机遇。第三，我们近年来始终注重“打基础、利长远”，狠抓招商引资和项目建设，培育了一大批新的增长点，经济上升的空间和潜力仍然很大。今年新增销售收入过1000万元的工业企业就有68家。鄂钢宽厚板一期、鄂州电厂二期、鄂州球团厂二期将先后投产；富晶电子、比克电池、浙江宝业、嘉吉烯王等一批过亿元的招商引资项目即将竣工投产，这些都为全市经济持续稳定发展奠定了良好基础。第四，鄂州是武汉城市圈“两型社会”综合配套改革试验区的重要城市，是全省城乡一体化试点城市，已经站在一个更高的发展平台上，这必将使鄂州在全省经济增长格局中的地位和作用越来越重要。只要我们审时度势，趋利避害，抢抓机遇，乘势而为，就一定能从容应对挑战，牢牢把握科学发展的主动权，继续保持经济社会平稳较快发展。

　　做好今年政府工作的总体要求是：认真贯彻党的十七大、十七届三中全会、省委九届五次全会和全市经济工作会议精神，以科学发展观统领全局，以“保增长、惠民生”为主线，围绕实现“两个率先”和建设“两型社会”先行区，着力推进投资增长和环境建设，着力推进改革创新和扩大开放，着力推进结构升级和发展方式转变，着力推进城市功能提升和民生改善，促进经济社会又好又快发展。

　　今年经济社会发展的主要预期目标是：生产总值增长13.5%左右；地方一般预算收入增长18%左右；全社会固定资产投资增长30%；规模以上工业增加值增长23%；全社会消费品零售总额增长16%；外贸出口增长12%；招商引资总额增长35%以上；实际利用外资增长12%；城镇居民人均可支配收入增长11%左右；农民人均纯收入增长10%左右；城镇新增就业2万人，城镇登记失业率控制在4.5%以内；人口自然增长率控制在6‰以内；单位生产总值综合能耗下降4.8%左右，二氧化硫排放量下降1.5%，化学需氧量排放量下降7.5%；居民消费价格涨幅与全省平均水平基本保持一致。

围绕以上目标任务，今年我们将着力抓好以下重点工作。

　　（一）坚持不懈地推进投资增长，积极有效应对全球金融危机。将保增长作为第一任务，将抓投资作为应对金融危机的第一措施，力争今年固定资产投资达到220亿元。全力以赴抓投资。重点协调和落实100个亿元以上项目。加快在建项目建设，确保竣工投产一批。着力抓好鄂钢宽厚板、鄂州电厂二期、球团厂二期、世纪新峰二期等30个亿元在建重点项目，实现今年竣工投产；扎实抓好前期工作，确保开工建设一批。着力抓好武鄂黄（黄）两条城际铁路、鄂州火车站、汉鄂快速通道、城市中轴线等基础设施项目和武钢焦化、华中数控、星丰废金属综合利用、程潮铁矿新选矿厂、科贝实验室设备等已具备开工条件的重点项目，力争今年新开工建设40个亿元重点项目；紧盯国家政策动向，超前策划储备一批。重点是抓好鄂州电厂三期、葛店开发区职教园、鄂东农产品批发市场、洋澜湖水环境治理和生态修复等30个亿元以上项目。进一步强化项目协调服务，继续实行重大项目市级领导联系制，定期召开重点项目调度会，及时帮助项目单位解决资金、征地、拆迁等困难和问题，为项目建设创造一流的外部环境。全力以赴引资金。进一步创新招商方式，大力提高招商效率。加强城市和开发区（园区）整体形象的策划、包装和推介，扩大知名度，提升竞争力；加强重大项目的前期工作，努力增强对投资基金和企业的吸引力。增加招商专班人员和经费，对中介人员和有功人员给予重奖，继续选派干部到发达地区挂职招商。积极探索顾问招商、委托招商、网上招商、业主自主招商、以商招商等新方式，赴长三角、珠三角和环渤海地区开展招商活动，努力提高洽谈项目签约率和签约项目开工率。紧盯世界500强、国内500强和民营500强企业，力争在引进重大项目和战略投资者上取得突破。全力以赴争项目。力争中央、省级各类投资12亿元，比2008年增长30%以上。抓好申报和争取国债项目工作，加快落实已申报的30个项目，对策划的130个国债项目实行专班跟踪，责任到人，力争更多的项目进入省和国家计划。抓住国家和省加大对工业企业技改进行贴息的机遇，加强政策研究和项目申报，促进更多的项目进入国家补贴范围。

　　（二）坚持不懈地推进结构调整，打造更具竞争力的产业体系。进一步提升新型工业化水平。大力改造提升冶金、建材、服装等传统支柱产业。加快鄂钢设备大型化改造、鸿泰钢铁、世纪新峰、顾地、鄂重改扩建等重大技改项目进度，支持鄂钢、鄂州电厂、世纪新峰、顾地、鄂重、多佳等一批企业做大规模，做强品牌，带动支柱产业整体提升素质。大力发展新兴产业，促进生物医药、电子信息、船舶工业等产业加速发展，使其逐步成为新的支柱产业。大力培育产业集群，加快表面处理工业园、太阳能产业园、浙江工业园、华工科技产业基地、武汉大学地理空间信息产业基地的争取和建设。对成长性好的燕矶金刚石刀具、葛店水泵铸造、梁子湖非金属加工等产业集群，实施政策倾斜，使其加速跨入10亿元产业集群行列。进一步加快发展现代服务业，大力提升传统服务业。落实扶持政策，重点发展旅游产业、现代物流业、中介服务业、信息服务业等现代服务业。围绕“一都两湖”，重点打造三国吴都文化游、梁子湖休闲度假游、红莲湖运动健身游等精品旅游线路，着力推进“景区创A、宾馆创星、导游创佳”，继续举办第十届梁子湖捕鱼旅游节。加大专业市场建设，有计划改造现有专业市场，加快新型专业市场建设步伐，进一步拓宽消费领域，改善消费环境。大力发展社区商业、物业、家政等服务业。抓好“万村千乡市场工程”，推进“农改超”试点，加强农超对接，切实做好“家电下乡”工作。调整出口商品结构，扩大外贸出口。进一步扶持中小企业发展。筹集1500万元，注入中小企业担保公司，使其资本金达5000万元，做大担保平台。支持金融机构在梁子湖区、花湖工贸新城设立分支机构，鼓励股份制银行到鄂州开设分支机构，给予免费提供办公场所和财税政策优惠等扶持。抓住当前货币政策适度宽松的机遇，组建1-2家小额贷款机构，规范融资行为，防范和化解金融风险，促进金融机构扩大对中小企业的信贷投入；完善中小企业成长工程的考核奖励办法，落实关于促进中小企业发展的系列措施，扶持一批成长性较好的规模以下企业，通过技术改造、扩大规模跨入规模以上企业行列，力争2009年规模以上企业达到460家。选择重点企业积极申报“湖北省著名商标”，力争今年达到20件，支持“鄂重”、“恒工”申报“中国驰名商标”。支持7家具备条件企业上市。毫不动摇地鼓励、支持、引导民营经济健康发展，扩大民营资本参与经营性基础设施和公共服务的范围，重点扶持一批规模型、科技型、外向型民营企业，在用地、融资等方面予以倾斜，切实改进对民营企业的服务，在全社会营造重视和支持民营企业发展的良好氛围。

　　（三）坚持不懈地推进城乡一体化，加快实现城乡统筹发展。全面贯彻党的十七届三中全会精神，进一步加快新农村建设步伐，推动城乡规划、产业布局、基础设施、社会保障、公共管理、市场体系六个一体化向纵深发展。打造城乡一体支撑平台。切实加大小城镇和新村建设力度，引导社会资金向农村流动。编制完成全市特色镇总体规划、中心村布点规划和近期建设试点村详细建设规划。加快推进特色镇、中心村建设，继续筹资6000万元支持十个特色镇建设产业园，筹资2000万元建设100个中心村。在中心村规划建设上实行分类指导，对人居环境好、历史文化深厚、不宜归并搬迁的村，统一规划建设为田园村、生态村、文化村等特色村。对零星布局、人口少的弱村、小村、偏远村，有计划、有步骤地实施迁村腾地、合村并居，改造为新社区。实施城乡一体重点工程。通过推动重点工程建设，大力改善农村生产生活条件。实施国土整理和高产农田建设工程，制定土地整理和高产农田建设总体规划；实施水系治理生态修复工程，编制水系治理生态修复规划，策划一批项目进入中省规划和项目库，争取梁子湖水系治理进入国家项目库；实施农村绿化美化工程，抓好退耕还林后续产业项目，鼓励农村种花植树，努力使全市森林覆盖率增加1个百分点；开展“清洁乡村、美化家园”活动，逐步推动农村垃圾集中收集、集中处理；逐步推动城市公交进城入镇试点，政府实行公交补贴，逐步实现市域内公交一体化营运；实施农业产业化龙头工程，培育和引进大中型农产品龙头加工企业，力争引进1-2家全国有影响的农产品加工龙头企业进驻鄂州。夯实城乡一体产业基础。以提高农民收入为主线，大力发展现代农业，夯实城乡一体的产业基础。切实抓好农业板块建设，着力推进水产、蔬菜、畜牧、林果四大支柱产业加快发展。切实抓好农产品质量安全工作，成立农产品质量安全检验监测中心，建立统一的农业投入品安全使用制度。深化集体林权制度改革，稳步推进第二轮整村扶贫开发。积极发展休闲农业、观光农业，进一步拓宽农民增收渠道。建立城乡一体推进机制。按照统筹城乡发展和建设社会主义新农村的总体要求，以破除城乡二元体制为重点，以转变政府职能、建设服务型政府为核心，筹建市统筹城乡发展委员会，理顺职责关系，创新体制机制，优化岗位设置和工作流程，全面履行领导和推进城乡一体化、新农村建设职责。深入推进乡镇机构改革，推动城乡管理有效衔接、行政管理与群众自治良性互动。巩固“以钱养事”成果，构建新型农业社会化服务体系。

　　（四）坚持不懈地推进改革创新，努力建设“两型社会”先行区。编制完成空间布局、产业发展、基础设施、社会事业、生态环境5个专项规划纲要，加强与省各项规划的对接，全面推进“两型社会”先行区建设。大力发展“两型产业”和循环经济。积极争取国家和省“两型产业”引导和扶持专项资金，大力发展循环经济。制定冶金、建材等重点领域循环经济专项规划，建立循环经济重大项目库，重点支持钢渣微粉深加工等10家循环型企业以及球团厂、鸿泰钢铁等10家企业建成清洁生产示范企业；配合武汉申报“青阳鄂”国家级循环经济高技术开发区，加快鄂州经济开发区省级循环经济示范区建设；加快鄂钢生产工艺循环、TRT发电、吴城钢铁水资源循环利用、城市垃圾资源化等循环经济项目的推进。积极推进各项改革试点。进一步优化政府组织体系和运行机制，按照决策权、执行权、监督权既相互制约又相互协调的要求，探索实行职责有机统一的大部门体制，全面完成市区两级新一轮机构改革任务。推动机构编制科学化、规范化、法制化管理试点，探索机构编制管理与财政预算、组织人事管理的配合制约机制。推行审批方式改革和行政事业收费改革，最大限度减少收费项目，逐步实行审批管理“零收费”制度。按照权责利相统一的要求，构建专业管理相对集中、综合管理重心下移、群众广泛参与的城市管理体制。深化政府投资工程和城市公用事业管理改革，建立规范、高效的管理机制。深入推进财政管理体制改革，全面推行“乡财区管乡用”改革和市直财政一级账户预算管理改革；进一步深化财政支出管理改革，全面实行公务卡，提高资金运行效率。切实加大节能减排力度。全面完成省政府下达的九个专项治理任务，制定并实施粘土砖厂淘汰计划和主城区水泥厂关闭、搬迁改造计划。加大巡查和在线监控力度，确保环保设施正常运行。抓好鄂钢高炉余压发电、烟气脱硫工程、鄂州电厂废水余能发电、世纪新峰余热烘渣、污水处理厂和垃圾处理厂等节能减排项目建设。切实抓好建筑节能，完善建筑节能监管体系，在主城区和三座新城所有新建居住建筑和公共建筑开展试点。

　　（五）坚持不懈地推进宜居宜业大城市建设，不断提升城市综合竞争力。全面提升城市功能。主城区按照“一体两翼”发展布局，抓紧编制市政基础设施建设规划、城东新区分区规划、三江港区建设规划以及各项专项规划。加快城市基础设施建设，计划投资15亿元完善城市功能，基本形成大城市框架。加快城西新区（鄂州经济开发区）建设，拉开西区主骨架，实施发展大道东段、双港大桥、青天湖路、滨港路等路桥新建工程，建设樊口污水处理厂管网工程；启动城东新区建设，启动城市中轴线东段工程，支持鄂城区机关办公区、生活区向城东新区整体搬迁，拉动城东新区加速发展；完善中心区城市功能，实施迎宾大道、寿昌大道、文苑路等道路续建工程，滨湖东路、武昌大道东段及武昌大道西段、沿江大道北门段等刷黑改造工程，三国吴都旅游风光带二期、洋澜湖生态公园一期及园林绿化、人行道改造和市政设施维护改造工程，城区背街小巷改造工程，垃圾处理场、标准化公厕等环卫设施建设工程，供水设施建设工程。加快建设三座新城。葛华科技新城投资5.7亿元，启动高新东路新建、高新西路扩建和创业园3号路、10号路等工程。红莲湖旅游新城投资2亿元，建设主干道、环湖路和污水管网等项目。花湖工贸新城投资1亿元，建设华山大道、胄山路、杭州路、环城路、友谊路北段和胄山桥工程。突破性发展城市交通。加速建设汉鄂快速通道，全面启动316国道改造、葛湖一级公路建设，打通红莲湖至高新六路、高新四路东段、六十至凤凰山等三条连接武汉的通道，全力做好武鄂黄（黄）两条城际铁路、武九线电气化改造等重大工程的协调工作，加快鄂州火车站改造工程建设，抓紧做好武汉新港三江港区专业规划设计和鄂钢货运码头工程建设，努力把鄂州建成鄂东交通枢纽城市。全力推进“四城”创建。今年是“四城创建”达标验收的关键年。坚持通过项目建设推进创建工作，对已确定的洋澜湖生态公园、西山公园、葛山森林公园、雷山植物园、三国吴都博物馆等43项重点工程，落实项目规划设计方案和建设方案，确保实施进度。加大舆论宣传和组织引导，动员机关、企事业单位和市民自觉参与“四城”创建，努力夯实创建基础，全面提升创建水平。继续深化城市管理体制改革，提升城市管理水平。努力促进房地产市场健康发展。加大保障性住房建设力度，通过新建、改造、收购等渠道，扩大保障性住房供给，解决好城市低收入住房困难家庭住房问题。全面落实国家关于住房消费的有关信贷和税费政策，改善住房供应结构，鼓励普通商品住房消费，扩大商品房交易量，降低房屋空置率。全面推行专业化、市场化物业管理，切实解决住宅小区水电直供问题。加大城中村改造力度，确保已启动的项目全部开工。

　　（六）坚持不懈地改善和保障民生，努力构建和谐鄂州。进一步稳定和扩大就业。推进全民创业，以创业带动就业。进一步放宽市场准入，鼓励自主创业，全面落实税费减免、小额担保贷款、资金补贴等政策，启动劳动密集型企业贷款贴息，开展创业培训，设立创业扶持资金，建立创业孵化基地，完善创业服务体系。进一步做好农村劳动力转移培训、困难群众就业、零就业家庭就业工作。加强技能培训，改进就业服务，做到求职用人信息对接。切实做好高校毕业生、退役军人就业工作。充分发挥失业保险调控和促进就业的功能，积极开展援企稳岗工作，保持就业局势基本稳定。进一步完善城乡社会保障体系。继续做好社会保险扩面征缴工作。完善和落实城乡居民最低生活保障制度，继续增加企业退休人员养老金。争取启动新型农村社会养老保险试点，加强对城乡困难群众的社会救助。整合城镇职工基本医疗保险、城镇居民基本医疗保险、新型农村合作医疗制度，做好关闭破产企业退休人员参加医疗保险工作，启动大学生医疗保险工作，实现医保全覆盖。全面启动非公有制企业住房公积金征缴工作，努力改善住房困难群众的居住条件。做好残疾人、农村五保户、下岗职工、失业人员等城乡困难群众的救助工作，探索实施农村失地农民安置保障的有效办法。努力保持物价稳定。进一步加快社会事业发展。支持鄂州大学发展，加快葛店职教园区规划和建设步伐，有效整合城乡义务教育资源，对农村中小学布局调整进行科学规划，集中资金完成全部农村初中寄宿制学校建设，积极稳妥地落实义务教育学校教师绩效工资政策。高度重视人才工作，加大招才引智力度。全面启动市中心医院、二医院、中医院、妇幼保健院改造项目，完成城区2家社区卫生服务中心和6家乡镇卫生院改造，积极稳妥地推进医药体制改革。继续抓好“百镇千村”乡镇综合文化站、农家书屋工程建设，抓紧筹建市综合体育馆；加强文化市场监管，广泛开展群众性文化体育活动，丰富市民精神文化生活。加快推进农村特困户危房、土坯房和四级敬老院改造工程。继续稳定低生育水平，加大出生人口性别比综合治理工作力度。进一步抓好安全生产和社会稳定。牢固树立“安全发展”理念，进一步落实安全生产责任制，坚决遏制重特大事故；加强食品药品安全监管，严格控制重大食品药品安全事故发生。健全信访工作责任制，妥善处理人民内部矛盾，及时发现和解决各类矛盾纠纷。完善应急反应机制，加强预警预测，积极做好抗灾救灾避灾工作。健全社会治安防控体系，积极推进平安创建活动，依法打击制毒贩毒和组织传销等违法犯罪活动，增强人民群众安全感。扎实推进援疆工作。积极开展抗震救灾对口支援，全面完成省委、省政府交办的各项援建任务。进一步加强社会管理。创新社会管理体制机制，积极发挥各类社会组织的作用。完善和加强城市社区功能。发挥工会、共青团、妇联等人民团体桥梁纽带作用，积极参与社会管理和公共服务。加强基层民主政治建设，健全政务公开、厂务公开、村务公开等制度。扎实开展国防动员工作，支持驻鄂州解放军和武警部队建设，不断提高民兵预备役工作质量和人民防空建设水平。积极开展双拥共建活动，巩固和发展军政军民团结。强化司法公正，加大对弱势群体的法律援助，支持审判机关和检察机关依法独立行使审判权和检察权。扎实开展“五五”普法工作。加强外事、对台、侨务、民族、宗教工作。继续做好统计、气象、地震、档案、史志等工作。重视和支持老龄、残疾人事业。全面繁荣新闻出版、广播电视、文学艺术、社会科学事业。

　　（七）坚持不懈地办好“十件实事”，不断改善市民生产生活条件。围绕解决群众最关心、最直接、最现实的利益问题，努力办好“十件实事”：（1）改善农民生活条件。筹资4000万元，再解决80个村10万人饮用自来水问题；筹资3060万元，建设大中型沼气工程4处，建成农村户用沼气池8000口；筹资610万元，对水稻、奶牛实行农业保险，对农村军属烈属、农村五保户、低保户实施住房保险。（2）改善农业生产条件。筹资1.56亿元，实施5万亩高产农田建设示范工程和4.1万亩低丘岗地改造项目；筹资700万元，对农民购置农机实施财政直补。（3）实施老城区水改电改入户工程。多渠道筹资4800万元，在老城区实施水、电入户改造各1万户。（4）提高城乡医疗服务能力。筹资1.5亿元，加大市中心医院、中医医院、妇幼保健院、二医院医疗基础设施建设；筹资1318万元，建设农村村级卫生室36个、乡镇卫生院5所、社区卫生服务中心2处。（5）丰富市民科技文化体育生活。筹资900万元，建设鄂州市职工活动中心；筹资200万元，新建100家农家书屋；筹资100万元，新建和改建6家乡镇综合文化站；启动建设市科技馆、体育馆。（6）继续改善城镇基础设施。筹资2000万元，继续对城区30条背街小巷进行改造；筹资6000万元，加快十个特色镇基础设施建设；筹资2000万元，实施100个中心村新村建设工程。（7）逐步缓解城镇低收入家庭住房困难。筹资1000万元，新建200套廉租房和经济适用房；筹资300万元，对2000户城市低收入住房困难家庭实行住房租赁补贴。（8）美化净化生活环境。筹资1000万元，实施洋澜湖清淤换水工程；实施主城区粉尘、烟、气、噪声污染治理工程。筹资8600万元，启动鄂州球团厂污染综合治理工程；筹资3000万元，启动鄂钢污染综合治理工程；关停10家“五小企业”。（9）改善农村办学条件。筹资2800万元，按规划布局完成22所农村初中寄宿制学校建设。（10）扩大城乡就业渠道。筹资1000万元，培训转移农村劳动力2万人；新增500万元小额担保贷款基金规模，支持全民创业。

　　三、切实加强政府自身建设

　　深入开展学习实践科学发展观活动，按照“用心做事、公正待人、发展为先、人民至上”的总体要求，以非常之作为应对非常之时期，建设一个为人民服务、对人民负责、让人民满意的政府。

　　努力提高科学发展能力。切实加强学习调研，认真进行分析检查，着力开展整改落实，始终保持开拓进取、奋发有为的精神状态，争做学习实践科学发展观的表率。用新视点发掘新优势。辩证看待版图小、人口少的市情，以“小”求“优”，以“小”谋“大”，争做改革创新大市、经济发展强市和城乡一体化城市。用新理念谋求新发展。立足应对危机，着眼长远发展，坚持走“绿色、集约、高端化”发展之路。在发展速度上力求率先，努力走在全省前列；在发展路径上力求转型，努力提升发展层次；在发展格局上力求统筹，努力营造和谐平安的社会环境；在发展宗旨上力求惠民，努力做到发展为了人民，发展依靠人民，发展成果由人民共享。

　　努力提高应对危机能力。切实提升危中求机的能力。坚持用宽广视野考量问题、用战略眼光谋划发展、用创新思维破解难题，咬住目标不放松，求真务实，真抓实干，把制定的规划、方案变成具体实在的项目，推动鄂州在危机之年又好又快发展。切实提升处理复杂矛盾的能力。以对党和人民高度负责的态度，深入基层、深入实际、深入群众，高度重视和妥善解决城镇发展、项目建设等工作中产生的矛盾和问题，做到排查矛盾保持常态，调处矛盾把握主动，化解矛盾立足长远，坚持用解决矛盾的办法维护社会稳定。切实提升促进协调发展的能力。进一步明晰思路、强化措施、勇于突破，既要实现经济平稳发展，又要推进社会全面进步；既要注重眼前利益，又要注重可持续发展。努力做到经济发展与环境保护同步，城市建设与村镇建设协调，保增长与惠民生兼顾。

　　努力提高对外开放能力。化解危机，加快发展，必须以更大的气魄和力度对外开放。进一步增强敢为人先、敢破常规的勇气。以解放思想为先导，增强创新意识，真正做到从实际出发，把握好原则的坚定性与政策的灵活性的辩证统一，创造性地贯彻落实上级方针政策，敢闯敢冒敢试，用切合鄂州实际的办法解决发展中出现的新情况新问题。树立拼搏有为、争当第一的观念，真正做到志存高远，尊重科学、尊重规律，从更广的领域、更高的定位来审视自己，敢于干大事、创大业、争一流。进一步提高走出去、引进来的本领。增强机遇意识、市场意识，大胆走出去，主动出击，逆势而上，用百分之百的努力争取百分之一的机会，广泛接触客商，深入洽谈项目，扎扎实实推进招商引资。坚持“走出去”与“引进来”同时并举、相互促进，努力在新一轮国际分工合作和经济结构调整中取得有利地位。进一步培育海纳百川、开放包容的城市精神。继续加大投资环境整治力度，精心营造亲商安商环境，满腔热情地接待和帮助外来投资者、打工者、旅游者和移居鄂州的建设者，使所有在鄂州工作和生活的人对鄂州都有认同感和归属感。

　　努力提高依法行政能力。切实做到依法行政。以推动政府科学决策、民主决策、依法决策为重点，进一步优化行政运行流程，完善行政绩效考核，强化行政问责机制，切实加强依法行政体系建设。进一步完善重大事项集体决策、专家咨询、社会公示与听证制度，不断提高政府决策透明度和公众参与度。自觉接受人大及其常委会的法律监督和工作监督，及时报告工作，落实各项决议、决定；自觉接受政协的民主监督，认真听取民主党派、工商联和无党派人士的意见，定期向政协通报工作情况；认真办理人大代表建议和政协委员提案，支持民主党派参政议政，自觉接受舆论监督。切实做到勤勉善政。巩固提高政府执行力建设成果，建设一支政治坚定、业务精通、作风优良、善于服务、甘于奉献的公务员队伍。深入体察民情，凡是事关发展和民生的重大事项，按照特事特办、急事急办的原则，狠抓落实。多做打基础、利长远的工作，多做破难题、解民忧的事情，创造经得起历史、实践和群众检验的实绩。切实做到清廉从政。严格落实党风廉政建设责任制，切实加强反腐倡廉建设；坚决纠正损害群众利益的不正之风，严肃查处各类违法违纪案件；强化审计监督、行政监察和行政层级监督，加大政府投资项目审计力度，实行审计结果公开通报制度，树立清正廉洁的政府形象。切实做到厉行节约、勤俭办事，大力压缩开支、节俭费用，精简会议，把更多的资金用于发展经济和改善民生。

　　各位代表，鄂州的发展已经进入了一个新的关键时期，新的一年充满挑战和希望，新的任务艰巨而繁重。人民群众对政府的信任和支持，给了我们无穷的力量和必胜的信心。让我们更加紧密地团结在以胡锦涛同志为总书记的党中央周围，认真贯彻落实省委、省政府的决策和部署，在市委的坚强领导下，深入贯彻落实科学发展观，坚定信心，团结奋进，勇于创造，扎实努力，在推进“两个率先”、建设“两型社会”先行区的征程上不断取得新的更大胜利，以优异成绩迎接建国六十周年！
